# Aging Field Collected *Aedes aegypti* to Determine Their Capacity for Dengue Transmission in the Southwestern United States

**DOI:** 10.1371/journal.pone.0046946

**Published:** 2012-10-12

**Authors:** Teresa K. Joy, Eileen H. Jeffrey Gutierrez, Kacey Ernst, Kathleen R. Walker, Yves Carriere, Mohammad Torabi, Michael A. Riehle

**Affiliations:** 1 Department of Entomology, University of Arizona, Tucson, Arizona, United States of America; 2 Research Computing Principal, University of Arizona, Tucson, Arizona, United States of America; 3 College of Public Health, University of Arizona, Tucson, Arizona, United States of America; University of Texas Medical Branch, United States of America

## Abstract

*Aedes aegypti*, the primary vector of dengue virus, is well established throughout urban areas of the Southwestern US, including Tucson, AZ. Local transmission of the dengue virus, however, has not been reported in this area. Although many factors influence the distribution of the dengue virus, we hypothesize that one contributing factor is that the lifespan of female *Ae. aegypti* mosquitoes in the Southwestern US is too short for the virus to complete development and be transmitted to a new host. To test this we utilized two age grading techniques. First, we determined parity by analyzing ovarian tracheation and found that only 40% of *Ae. aegypti* females collected in Tucson, AZ were parous. The second technique determined transcript levels of an age-associated gene, Sarcoplasmic calcium-binding protein 1 (*SCP-1*). *SCP-1* expression decreased in a predictable manner as the age of mosquitoes increased regardless of rearing conditions and reproductive status. We developed statistical models based on parity and *SCP-1* expression to determine the age of individual, field collected mosquitoes within three age brackets: nonvectors (0–5 days post-emergence), unlikely vectors (6–14 days post-emergence), and potential vectors (15+ days post-emergence). The statistical models allowed us to accurately group individual wild mosquitoes into the three age brackets with high confidence. *SCP-1* expression levels of individual, field collected mosquitoes were analyzed in conjunction with parity status. Based on *SCP-1* transcript levels and parity data, 9% of collected mosquitoes survived more than 15 days post emergence.

## Introduction

The worldwide incidence of dengue has increased steadily since the 1960's. Currently, an estimated 100 million people are infected annually and serious outbreaks are becoming more common [1], [2]. All four serotypes of dengue are now found in the Americas, Asia and Africa, which greatly increases the risk of dengue hemorrhagic fever (DHF) [1]–[5]. Furthermore, the worldwide direct and indirect costs of dengue infections amount to billions of dollars annually [6]. In the Americas, estimates range from one to three billion dollars annually depending on frequency and severity of outbreaks. A majority of these costs are due to lost productivity, as many infections prevent people from working for a week or more [6].

Local transmission of dengue in the continental United States has occurred in the border towns of Brownsville, Texas and Matamoros, Chihuahua in 2005 and Key West, Florida and Miami-Dade County, FL in 2010 and 2011 [7]–[9]. Another likely route of dengue entry into the United States is through the southwest border, including Arizona. Like the sites in Texas and Florida, southeastern Arizona has well established populations of *Aedes aegypti*, the primary vector of dengue and DHF. Furthermore, dengue is endemic in the Mexican state of Sonora which borders Arizona. Since the 1990's Hermosillo, Sonora, just 178 miles south of the Arizona/Sonora border, has experienced seasonal dengue transmission and several large outbreaks [10]. Arizona/Sonora border towns are major hubs of travel, immigration, and trade with Mexico, increasing the likelihood of dengue introduction into the United States through southern Arizona.

Even though *Ae. aegypti* can be found throughout urban areas of the Southwestern US including Nogales, Tucson, and Phoenix, local transmission of dengue has yet to be demonstrated. Multiple factors must be in place for dengue to emerge in a given region; dengue virus must be introduced into the population, interactions between a competent vector and susceptible human population must be present and introduction must coincide with the *Ae. aegypti* season. In the southwestern United States there may be limited water sources for oviposition decreasing overall vector density and high quality housing may reduce contact with the vector minimizing but not eliminating transmission potential, [11]. Another contributing factor could be that the hot, dry climate of southeastern Arizona, which represents the northern edge of the mosquito's range, prevents *Ae. aegypti* from surviving long enough to effectively transmit the virus. Even relatively small reductions in lifespan can significantly impact transmission potential given the exponential relationship between vectorial capacity and survival.

Following an infective bloodmeal, the dengue virus must complete an extrinsic incubation period (EIP) averaging 10–14 days in the mosquito vector before it can be transmitted to a human host. This EIP is temperature dependent, and has been shown to increase as temperatures decrease [12]. At high temperatures (≥32°C) the EIP of dengue can be as short as seven days. At 30°C the EIP was found to be as long as 12 days. Mean temperatures in Tucson, AZ during the peak of *Ae. aegypti* activity range from 30.5°C in July to 27.7°C in September [13], suggesting an EIP in our study area of ∼12 days. Since host seeking in *Ae. aegypti* does not begin until 36–48 h post emergence [14], mosquitoes in the study site are unlikely to be dengue vectors until they are ∼14 days old.

The above averages, however, mask great diurnal variability and temperatures frequently range 10°C over the course of the day in Tucson. Average high temperatures reach 37.6°C in July and 34.7°C in September, with temperatures often exceeding 40°C [13]. Extreme high temperatures such as this have a great impact on adult survivorship. For example, 50% of adult *Ae. aegypti* perish in less than an hour when maintained at 40°C [15]. Mark-release-recapture studies show the adult lifespan of *Ae. aegypti* to be relatively short, although in dengue endemic areas a significant portion of the population does survive the EIP. In some urban neighborhoods of Rio de Janeiro where dengue is endemic, as many as half of mosquitoes survived more than ten days post-emergence during the dry season, while approximately a third survived the ten day EIP during the wet season [16]. In contrast, suburban neighborhoods in Rio de Janeiro had fewer mosquitoes surviving the 10 day EIP with the first having approximately 10% of the *Ae. aegypti* population surviving ten days and the second having 4% to 26% survivorship after ten days [16], [17]. Finally, a mark-release-recapture study in a high income, low density neighborhood in Rio de Janeiro found the 10 day survivorship was only between 0.6 and 13% [18]. Dengue transmission intensity was highest in neighborhoods with higher survival rates but this may be confounded by other factors that differed between these neighborhoods. Studies conducted by McDonald (1977) [19] in Mombasa, Kenya predicted daily survivorship to be 89%, meaning that approximately 19% to 31% of female *Ae. aegypti* would survive the 10 to 14-day EIP. Daily survivorship of *Ae. aegypti* ranged from 86 to 91% in northeastern Australia, where sporadic dengue outbreaks occur, indicating that 12 to 27% of the population could survive a 14-day EIP [20]. These survival rates from endemic areas suggest that transmission can be well established when as little as 10–27% of the *Ae. aegypti* population survives past the 10–14 day EIP. However, estimating the age structure of wild mosquito populations is difficult. Mark-release-recapture studies are valuable to determine mosquito survivorship, but these studies are laborious, difficult to conduct in some locations, and often use laboratory-reared mosquitoes that may not accurately represent wild mosquito populations. Such studies also tend to assume that mosquito survivorship is constant, although Styer et al. (2007) found that the mortality rate increased with age [21]. The ability to rapidly determine the age structure of wild *Ae. aegypti* populations in endemic and non-endemic areas would allow us to better understand how adult survivorship impacts dengue transmission.

Several techniques have been developed to determine the chronological or physiological age of arthropod vectors. Physiological age grading techniques are largely based on morphological changes that mosquitoes undergo during different developmental stages and physiological processes such as reproduction. These include the dissection of mosquito ovaries and analysis of ovarian tracheae to determine the parity status of individual females [22], counting ovarioles to approximate the number of egg clutches a female has laid [23], and counting growth lines on the insect cuticle [24], [25]. The first technique, tracheae analysis, provides the most reliable indicator of parity and is logistically easier than the second and third techniques [26].

Three well documented techniques have been used to determine a mosquito's chronological age and whether they can survive a pathogen's EIP: gas-liquid chromatography to determine the composition of cuticular hydrocarbons, near-infrared spectroscopy (NIRS), and gene transcription [27]–[34]. The analysis of cuticular hydrocarbon composition in *Ae. aegypti* was shown to accurately age mosquitoes up to 15 days post-emergence [29], [30]. However, to estimate the risk of dengue transmission it is important to accurately identify those mosquitoes older than 15 days post emergence, as these old mosquitoes are the primary vectors of the virus. In addition, expression of cuticular hydrocarbon abundance has been shown to change as the ratio between n-alkanese and n-alkene shifts according to external temperatures. NIRS has been shown to be cost effective after the initial purchase of the equipment, and not as time consuming as many other methods [27], [28]. One drawback is that considerable effort is required to calibrate models for particular mosquito species before using the technique on a large scale, although once this is accomplished subsequent analysis is rapid and inexpensive. It is also not possible to store field collected mosquitoes for extended periods of time before analysis. Finally, at this time NIRS can only differentiate female mosquitoes into young (<7 d) and old (>7 d) groups. Gene transcription analysis may provide more accurate information on the chronological age of mosquitoes [31]–[35]. Microarray data from the fruit fly *Drosophila melanogaster* and mosquito *Anopheles gambiae* has paved the way for identification of age associated genes in invertebrates [33], [35]–[40]. *Ae. aegypti* orthologues of *D. melanogaster* and *An. gambiae* genes have been used to age mosquitoes in the lab and under field conditions [31], [32], [34].

Here we used two techniques to age *Ae. aegypti* populations in Tucson, Arizona. We analyzed ovary tracheae to establish parity and transcript analysis of the Sarcoplasmic calcium binding protein 1 (*SCP-1*) to assess age [31], [39]. These two techniques allowed us to accurately group individual wild mosquitoes into non-vectors (≤5 days post-emergence), unlikely vectors (6–14 days post-emergence), and potential vectors (≥15 days post-emergence).

## Materials and Methods

### Mosquito rearing

*Ae. aegypti* mosquitoes (UGAL strain) were reared and maintained at 27°C and 70% RH with a 16 h light and 8 h dark photoperiod. Larval mosquitoes were reared at a density of approximately 150 larvae per liter of water, and maintained on cat chow to ensure optimal growth. Adult mosquitoes were given access to a 10% dextrose solution *ad libitum*. Mosquitoes used in blood feeding assays were given access to porcine blood containing 1% sodium citrate (obtained from the University of Arizona Meat Science Lab; IACUC: 05-182; PHS Assurance #A3248-01). Blood meals were offered weekly until mosquitoes were collected from the final time point. Oviposition substrates were offered 48 h after each blood meal for 24 h.

### Characterization of age-associated genes

Female *Ae. aegypti* mosquitoes (10–15 individuals) that had not been previously blood fed were collected 1, 3, 5, 10, 15, 20, or 35 days post-emergence. Total RNA from the pooled mosquito samples was isolated using the RNeasy Total RNA kit (Qiagen, Valencia, CA). Total RNA was treated with DNAse (Fermentas; Thermo Scientific Inc., Glen Burnie, MD) to eliminate genomic DNA contamination and converted into cDNA using the High Capacity cDNA Reverse Transcription kit (Applied Biosystems, Carlsbad, CA). Quantitative real-time PCR (qRT-PCR) was performed using primers against eight previously identified age-associated genes (Table 1). Three of these genes, *SCP-1*/*Ae-15848*, *CG-8505/Ae-8505*, and *fizzy*/*Ae-4274*, and one reference gene *RPS17/Ae-RPS17* were identified in *Ae. aegypti* [31] and the primers used were identical to those previously reported. An additional five age associated genes, *AGAP009551*, *AGAP011615*, *AGAP002827*, *AGAP005501*, and *AGAP009790* were adopted from *An. gambiae* [33]. Putative orthologs of these genes were identified in *Ae. aegypti* and new primers were developed (Table S1). The *Ae. aegypti RPS17* gene was used as a loading control in qRT-PCR as described by Cook et al. (2006). The qRT-PCR reactions were performed using the Maxima SYBR Green/ROX qPCR Master Mix (2×) kit (Fermentas; Thermo Scientific Inc., Glen Burnie, MD) and a Mastercycler® ep realplex real-time PCR machine (Eppendorf, Hauppauge, NY) at the following cycling conditions: stage 1, 50°C for 2 min; stage2, 95°C for 10 min; stage 3, 95°C for 0:15 s, and 65°C for 1 min; for 40 cycles. If an age-associated expression pattern was observed, five additional replicates were combined to form a standard curve of gene expression with pooled mosquito samples.

**Table 1:** Age associated genes.

	Gene Title	Function	Expected Expression Change with age	df/F	P
**Cook et al. (2006)**	SCP-1/Ae-15848	Calcium binding protein	Decrease	df = 5, 36 F = 5.15	P = 0.0012
	CG/Ae-8505	Structural component of cuticle	Decrease	df = 6, 10 F = 1.23	P = 0.37
	Fizzy/Ae-4274	Cell cycle/cell physiology	Decrease	df = 6, 10 F = 0.66	P = 0.68
**Wang et al. (2010)**	AGAP009551	Sulfotransferase domain, response to stress	Increase	df = 6, 12 F = 1.05	P = 0.44
	AGAP011615	Chitin binding Peritrophin-A domain	Increase	df = 6, 56 F = 5.26	P = 0.0002
	AGAP002827	Synaptic vesicle membrane, transporter activity	Increase	df = 6, 13 F = 3.54	P = 0.026
	AGAP005501	NAD(P)(+)-binding proteins, oxidoreductase activity	Decrease	df = 6, 40 F = 0.67	P = 0.67
	AGAP009790	Chitin binding Peritrophin-A domain	Decrease	df = 6, 17 F = 1.86	P = 0.15

Nine previously established age-grading genes were tested on pooled mosquito samples of known ages (3, 5, 10, 15, 20, and 35 days post emergence). The gene *RPS17* was used as a loading control when running qRT-PCR on all nine genes. Three genes *SCP-1*/*Ae-15848*, *CG/Ae-8505*, *Fizzy/4274*, and the control *RPS17* were adopted from Cook et.al, 2006 [31] while six genes, *AGAP009551*, *AGAP011615*, *AGAP002827*, *AGAP005501*, *AGAP009790*, *AGAP007963* were adopted from *An. gambiae* genes identified by Wang et.al 2010 [33]. . A one-way ANOVA was performed on each gene, degrees of freedom, F-values and P-values are provided.

To verify that expression of the age associated genes was not influenced by reproductive status or environmental conditions, we replicated the above assays with lab-reared mosquitoes provided with multiple blood meals and mosquitoes reared under semi-field conditions, but not blood fed. Blood fed mosquitoes were provided with weekly blood meals and oviposition substrates (blood meal and eggs were extracted from each mosquito prior to processing). For semi-field studies adult mosquitoes were maintained in 5 L plastic cages (Rubbermaid #3922 modified with screen top and stocking net access) exposed to the Tucson, AZ environment. Cages were stored under a small shelter to protect them from direct sunlight and rain and mosquitoes were provided with a 10% sucrose solution. Semi-field studies were conducted from May to September of 2010, which corresponds to southern Arizona's monsoon season and represents the annual peak of *Ae. aegypti* activity in Tucson [41].

### Validation of age-associated gene expression using individual mosquitoes

To be of optimal use in the field, age grading individual mosquitoes, not just pooled samples, is essential. To analyze gene expression, 127 individual mosquitoes were collected at various adult ages (3, 4, 7, 8, 10, 12, 13, 14, 15, 19, 20, 24, 27, 30, 35 and 39 d after adult emergence) from multiple generations of lab reared mosquitoes. Total RNA was isolated and cDNA synthesized for each individual mosquito as described above, followed by qRT-PCR using *SCP-1* and *RPS17* primers as discussed above. The predicted age of the individual mosquitoes based on statistical analyses of SCP expression levels (see below) was compared with their known age. Twenty seven additional individual mosquitoes from these time points, reared under semi-field conditions, were also analyzed as described above for a total of 154 individuals.

### Collection of wild *Ae. aegypti* mosquitoes

A residential site in downtown Tucson, AZ was chosen for trapping mosquitoes. This area showed a high density of *Ae. aegypti* in a previous city wide survey using oviposition traps in 2004 [41]. Mosquitoes were trapped weekly, from July 20^th^ to October 3^rd^ 2009, using the BG Sentinel trap (BioQuip Products, Inc., Dominguez, CA). The trap was activated on Monday evenings and remained active until Friday evenings. Mosquitoes were collected from the trap twice daily, once in the morning before 8:00 am and once in the afternoon after 6:00 pm to minimize desiccation and mortality in trapped mosquitoes. Collected mosquitoes were individually labeled and stored at −80°C until analyzed for parity status and *SCP-1* transcript expression.

### Determining parity status and *SCP-1* transcipt levels of field collected mosquitoes

We determined the parity status of *Ae. aegypti* mosquitoes collected in the field in 2009 (N = 219) using a slightly modified version of previously established protocols [22], [26]. Briefly, ovaries were dissected directly into nanopure water droplets, rinsed with nanopure water, separated, and allowed to completely dry on a clean microscope slide. Parity status was determined by analyzing each dried ovary under an inverted microscope and observing trachea coiling. Images of each ovary were archived at 100× and 400× magnification using a Spot RT color camera (Diagnostic Instruments Inc., Sterling Heights, MI). Mosquitoes were scored as nulliparous if multiple trachea coils were observed on the surface of the ovary. Mosquitoes without coiled trachea indicating a previously completed reproductive cycle and gravid mosquitoes were scored as parous. Investigators scoring the ovaries first performed blind assays on mosquitoes of known parity until their accuracy rate was approximately 95%.

The remaining carcass (ie whole body minus ovaries) of field collected mosquitoes was processed for cDNA synthesis as described above. All cDNA samples were archived at −20°C for subsequent qRT-PCR assays. *SCP-1* expression levels were determined by qRT-PCR as described above. To compare the two approaches, the predicted age of the mosquitoes based on statistical analyses of SCP expression levels (see below) was determined for nulliparous and parous mosquitoes.

### Statistics

Statistical analyses were performed for all eight genes from the pooled mosquito trials. The effect of age on the C_t_ ratio (putative age associated gene/*RPS17*) from pooled mosquitoes was analyzed with a one-way ANOVA. Further analyses were performed on the *SCP-1* gene which showed the most consistent change with age. To assess whether *SCP-1* expression changed as mosquitoes aged, the C_t_ ratio (*SCP-1*/*RPS17*) from pooled mosquitoes was analyzed with a two-way ANOVA (JMP 2010). The response variable was the C_t_ ratio (log_10_ transformed), and explanatory variables were treatment (lab-reared non-blood fed, lab-reared blood fed, and semi-field reared non-blood fed mosquitos), age (treated as a categorical variable), and the interaction between these factors. As neither treatment nor the interaction significantly affected the C_t_ ratio (see Results), a two-way ANOVA without the interaction was fit, and Tukey tests were used to compare least squares means of the C_t_ ratio among mosquito age. Logistic regression was used to evaluate the association between *SCP-1* expression and age of individual mosquitoes to classify them based on *SCP-1* expression (SPSS 2010). Mosquito age was treated as a binary response variable (mosquitoes ≤14 days vs. >14 days). Preliminary analyses showed that the association between *SCP-1* expression and mosquito age did not differ significantly between lab and semi-field mosquitoes (lab vs. semi-field, Wald χ^2^ = 0.41, P = 0.52; experimental condition×*SCP-1* expression, Wald χ^2^ = 2.19, P = 0.14), but that a negative association between *SCP-1* expression and age of the mosquitoes occurred (Wald χ^2^ = 26.51, P<0.001). Lab and semi-field mosquitoes (N = 154) were thus pooled for analyses and the association between *SCP-1* expression and mosquito age evaluated. Three outliers were identified at this point. Due to our inability to find an error in collecting and processing these samples, each outlier was incorporated in the statistical analysis. Coordinates of the Receiver Operating Characteristic (ROC) curve and associated sensitivity and 1- specificity values were used to determine appropriate *SCP-1* expression cutoff points to classify mosquito age. A random sample of 62% (n = 96) of the observations was selected (Bernoulli distribution) to generate a predictive model. Accuracy of the predictive model was assessed using the remaining mosquitoes of known age (n = 58).

Mosquitoes may be nulliparous because they are too young to have reproduced, or older but were unable to find a blood meal. To use *SCP-1* values to better understand the origin of field collected nulliparous mosquitoes, a second logistic regression model was fit, using the binary response variable mosquito ≤5 days vs. >5 days. We did not use a random sample of the data to validate the *SCP-1* cutoff point resulting from this model because only 22 mosquitoes were ≤5 day old.

## Results

### Using *SCP-1* to determine the age of lab reared mosquito pools

**Figure 1:**
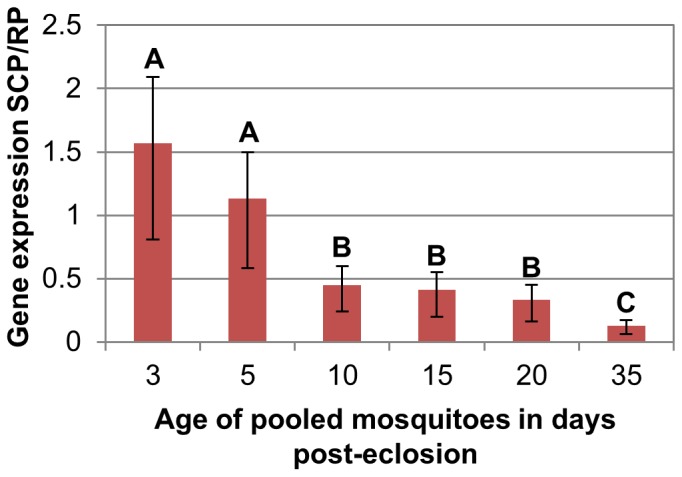
*SCP-1* gene expression in pooled mosquito samples of known ages. To evaluate effects of post emergence age and treatment on the C_t_ ratio (*SCP-1*/*RPS17*), groups of mosquitoes were compared using a two-way ANOVA followed by Tukey tests on least squares means. The means presented in this figure are untransformed. Different letters indicate significant difference in gene expression (p<0.05). Seven replicates were used for each time period in sugarfed, lab- reared mosquitoes; 3 replicates were used for each time period in bloodfed and semi-field reared mosquitoes. Each group consisted of 10–15 female mosquitoes. Bars indicate standard error.

Of the eight genes assayed (Table 1), the *Ae. aegypti* gene *SCP-1*/*Ae-15848* showed the most consistent change in expression as mosquitoes aged. Expression of *SCP-1* in pooled mosquitoes did not differ among lab reared, semi-field and blood fed lab reared mosquitoes (F = 0.20, P = 0.81), nor was the change in expression with age significantly different among these three groups (F = 0.42, P = 0.93). However, expression of *SCP-1* was significantly reduced in older mosquitoes (F = 18.71, P<0.001, Figure 1). Among-age comparisons revealed that *SCP-1* expression was highest in 3- and 5-day old mosquitoes, intermediate in 10-, 15-, and 20-day old mosquitoes, and lowest in 35-day old mosquitoes (Figure 1).

Two other genes *AGAP002827* and *AGAP011615* showed significant changes in gene expression with age (Figure S1).Expression of *AGA011615* was significantly higher in pooled mosquito samples in 35 day old mosquitoes (Figure S1D). Unfortunately, it was highly variable when we tested individual 35 day old mosquitoes instead of pooled mosquito samples (data not shown), making it unsuitable for age-grading older individual mosquitoes from the field. Expression of *AGAP002827* was significantly different in 1 day old mosquitoes (Figure S1E). However, discrimination of newly emerged mosquitoes is not useful in addressing dengue transmission potential, so further characterization of this gene's expression pattern was not pursued. The other five genes did not show significant changes in expression with age (Figure S1).

### Using *SCP-1* to determine age of individual mosquitoes

The logistic regression model for mosquitoes greater than 14 days of age (log odds of >14 day old mosquitoes = −3.69+17.37 (*SCP-1* expression), Likelihood Ratio χ^2^ = 31.98, P<0.0001) had a good fit (Hosmer–Lemeshow Chi-Square = 2.65, P = 0.95, Nagelkerke's R-square = 0.74) and an overall classification accuracy of 87.0%. Area under the ROC curve was large (area = 0.95, SE = 0.016, Asymptotic Significance <0.001). Ninety-one percent of mosquitoes ≤14 days had *SCP-1* values ≥0.46 (Sensitivity = 0.91), while 13% of mosquitoes >14 days had *SCP-1* values ≥0.46 (1 - Specificity = 0.13). Thus, using *SCP-1*≥0.46 as a cutoff point is expected to misclassify 9% of mosquitoes ≤14 days as being older and 13% of mosquitoes >14 days as being younger. Results from validation of the model with a random sample of the data show that the model predicted the age group 9 out of 10 times (overall classification accuracy = 90%; 80% for older mosquitoes; 97% for younger mosquitoes).

The logistic regression model for mosquitoes less than five days of age (log odds of <5 day old mosquitoes = 5.98−5.56 (*SCP-1* expression), Likelihood Ratio χ^2^ = 20.89, P<0.001) had a good fit (Hosmer–Lemeshow Chi-Square = 2.52, P = 0.96, Nagelkerke's R-square = 0.77) and an overall classification accuracy of 94.8%. Area under the ROC curve was 0.98 (SE = 0.009, Asymptotic Significance <0.001). Eighty-nine percent of mosquitoes ≤5 days had *SCP-1* values≥0.94 (Sensitivity = 0.89), while no mosquitoes >5 days had *SCP-1* values≥0.94 (1 - Specificity = 0). Thus, using *SCP-1*≥0.94 as a cutoff point is expected to misclassify 11% of mosquitoes ≤5 days as being older and 0% of mosquitoes >5 days as being younger. Using cutoff points from both logistic regression models, we were thus able to classify mosquitoes as ≤5 day old if *SCP-1*>0.94, 6 to 14 day old if 0.46<*SCP-1*≤0.94, and >14 day old if *SCP-1*≤0.46. Average *SCP-1* expression for these age classes is presented in Figure S2.

### Determining parity status of field collected mosquitoes

**Figure 2:**
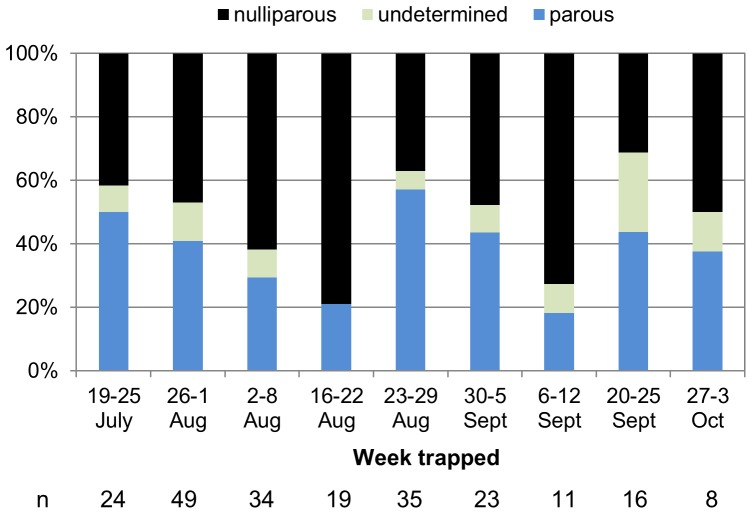
Parity status of field collected mosquitoes. The parity status of field caught mosquitoes was established by analyzing ovary tracheation. Individual mosquitoes were separated according to the week they were trapped. The parity status could not be determined for approximately 10% of mosquitoes (n = 21).

The percentage of trapped female mosquitoes that were parous averaged 40% (Figure 2) over the course of the season, ranging from 18% in the second week of September to 57% in the third week of August. There were 1.3 times more nulliparous mosquitoes (n = 110) trapped throughout the season than parous mosquitoes (n = 88). Included in the parous category were nine individuals (4.6%) with some degree of ovarian development. We were unable to confirm parity status of approximately 10% (n = 21) of the collected mosquitoes due to the poor state of the mosquito at the time of collection or damage/stretching of the ovaries during dissection (Figure 2).

### Determining age in field collected mosquitoes by combining gene expression and parity

**Figure 3:**
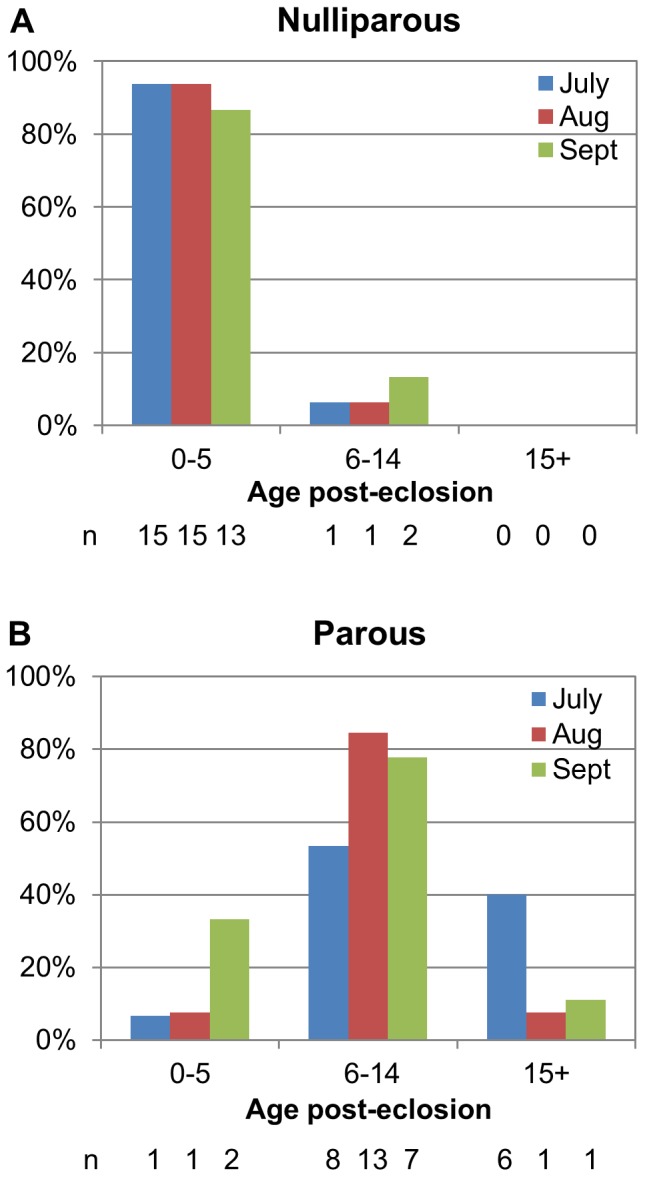
Predicted age of individual field collected mosquitoes of known parity. Based on cutoff points from logistic regression models, the C_t_ value of each mosquito was assigned into one of three catagories: 0–5 days post emergence, 6–14 days post emergence, or 15+ days accordingly. **A.** Nulliparous mosquitoes (n = 47) were randomly chosen from three, three-week time periods throughout the 2009 monsoon season. Based on cutoff points, all 47 nulliparous mosquitoes were less than 15 days post emergence, and 91% of them were less than 5 days post-emergence. **B.** Parous mosquitoes (n = 39) were randomly chosen from the same three time periods. Based on cutoff points, 79% of the parous mosquitoes were 14 days or less post emergence, while 21% were greater than 14 days post emergence.

A subset of nulliparous (n = 47) and parous (n = 39) mosquitoes were randomly selected to test for *SCP-1* expression. According to the cutoff *SCP-1* values determined in the logistic regression model, 100% of the nulliparous mosquitoes were less than 15 days post adult emergence (Figure 3a). The majority of nulliparous mosquitoes (91%) were found to be 5 days or less post adult emergence. In contrast, most of the parous mosquitoes (69%) were between 6 and 14 days post adult emergence. Only 10% of the tested parous mosquitoes were age graded as less than five days old, and only 21% of parous mosquitoes tested were found to be older than 14 days post adult emergence (Figure 3b). Combining the percentage of parous mosquitoes surviving at least 15 days with the average parity rate of 40%, we find that only ∼9% of the field collected mosquitoes in our sample survived long enough to be potential vectors of dengue.

## Discussion

The age structure of mosquito populations is a vital component in the vector's ability to efficiently transmit the dengue virus between human hosts. If insufficient numbers of mosquitoes survive the EIP of the virus, transmission is unlikely to occur. Aging individual wild mosquitoes can be used to validate outputs from models that use climatic inputs to predict the proportion of *Ae. aegypti* that survive the EIP. Incorporating data that quantifies mosquitoes older than the EIP is especially beneficial for modeling to predict the transmission potential of dengue in a given area. One such model, DyMSim, has been calibrated for use in both *Ae. aegypti* and *Culex quinquefasciatus* [42]. By comparing the monthly density of trapped mosquitoes to factors including regional geography and climate data such as temperature, relative humidity, rainfall, and solar radiation, the DyMSim model has been shown to accurately predict inter and intra-annual shifts in *Cx. quinquefasciatus* population densities [42]. If in addition to mosquito density, associations can be found between population longevity and climatic factors, models of vector competency and dengue risk across geographic regions will be estimated with increased accuracy.

From this research we determined the parity status of *Ae. aegypti* in Tucson, AZ based on ovary tracheation. Over the 2009 field season, we found that only 40% of trapped mosquitoes were parous or gravid, suggesting that most mosquitoes are very young or have decreased access to hosts compared to other areas. This parity level is similar to results from another study conducted in Tucson, in which 44% of female *Ae. aegypti* trapped between August and September were parous [43]. The percent of parous females trapped in Tucson is much lower than recorded percentages in some dengue endemic regions such as Rio de Janeiro, Brazil (92.9%) [16], [17], and in Trinidad, West Indies (99%) [44]. A recent study conducted in dengue-free Al-Madinah, Saudi Arabia found parity rates as low as 29.1% [45]. This would seem to support the hypothesis that the aridity of desert environments does not allow *Ae. aegypti* to survive long enough to transmit dengue, however dengue is endemic in the neighboring cities of Jeddah and Makkah, Saudi Arabia, illustrating the complex factors involved in dengue emergence [45].

Aging individual mosquitoes provides insight into age variation within a population. Thus, we validated the use of *SCP-1* to age individual mosquitoes, instead of the pooled samples. We generated two logistic regression models that together differentiated well between nonvectors (0–5 days post-emergence), unlikely vectors (5–14 days post-emergence), and potential vectors (15+ days post-emergence) based on *SCP-1* expression. Through the use of an established age grading technique (analysis of ovary tracheation) and *SCP-1* expression, we then determined that only 9% of the mosquitoes tested from our Tucson, AZ site were older than 15 days post emergence, suggesting that most *Ae. aegypti* individuals residing in Tucson may be too young to transmit dengue.

It is important to note that the age structure of the *Ae. aegypti* population is only one factor that influences transmission potential in Tucson, AZ. Factors directly associated with the vector including *Ae. aegypti* population density and vector competence also play a role, though *Ae. aegypti* from Tucson have been shown to be competent transmitters of DENV 2 [46]. Indirectly social factors may also play a significant role as modifiers of vector-human contact. As compared to endemic areas in low income countries, higher quality housing in the Arizona may prevent vector entrance into the homes while high temperatures may prevent individuals from frequent outdoor activity. Yet while social infrastructure has been associated with decreased intensity it has not completely prevented dengue emergence in the United States [11]. At the same time, human behaviors associated with dengue risk may also influence vector longevity. Endophilic mosquitoes in dengue endemic regions may also survive better when sheltered from the environment than those mosquitoes forced outdoors due to improved housing. Yet this does not explain why autochthonous transmission has not yet been reported south of the border in Nogales, SN, MX where climatic conditions are similar to Tucson, AZ but social infrastructure is similar to other endemic regions in northern Mexico. Future studies will further explore these factors.

This proof of principle study confirms the usefulness of combining two age grading techniques, *SCP-1* transcription and parity analysis. Not only can we accurately age single mosquitoes within the age groups, 1–14 days, or 15 days or more post emergence, but we can also identify mosquitoes less than 5 days post emergence. When coupled with parity analysis, the use of a single age-associated gene has proven to be a quick and efficient way to classify mosquitoes into these three age groups. A recent study by Hugo et al. (2010) [32] demonstrated that testing single mosquitoes on three genes (*SCP-1*/*Ae-15848*, *Ae-8505*, and *Ae-4274*) using a Taqman detection system aged 72% of single *Ae. aegypti* mosquitoes within ±6 days of their actual age. They also found no significant difference in *SCP-1* expression between mosquitoes released into the wild and reared in semi-field conditions [32]. These findings further validate the use of *SCP-1* gene expression in semi-field reared *Ae. aegypti* to approximate the age of field caught individuals.

The identification of additional age-grading genes, especially those with increased expression in older mosquitoes, would refine the accuracy of this technique. In testing previously described age-associated genes, we only found a consistent and significant pattern with SCP-1, in contrast to other studies [31], [32], [40]. One possible difference is that we isolated total RNA from the entire carcass minus the ovaries, whereas other studies utilized only the thorax and head. It is possible that variation in abdominal gene expression could have masked the age-associated expression patterns and we will explore this possibility in the future. In addition, microarray studies continue to be conducted on various mosquito species, introducing the possibility of identifying novel age-associated genes [33], [34], [40]. Recently, microarray studies have identified 35 genes in *Ae. aegypti* with age related gene expression [34], while 179 age related *An. gambiae* genes were not only found to be unaffected by one or more blood meals but 112 of the 179 genes showed a monotonic increase or decrease with age [33]. In the future it would be ideal to test more of these genes for similar expression trends in *Ae. aegypti*. The age grading genes that Wang et. al, 2010 [33] identified as homologous to *D. melanogaster* may be of special interest to future studies because like *SCP-1*, they can be applied to a greater number of arthropod species.

The next step in our research is to test the combination of the aging techniques, *SCP-1* gene expression and ovary tracheation, on a greater geographic scale across the southwestern United States and in nearby dengue endemic regions. Future testing of this set of techniques in endemic regions will reveal if differences in population ages exist between endemic and non-endemic areas. To establish the accuracy of age grading field caught mosquitoes with these techniques, one collection site was used during 2009. During the summer rainy seasons of 2010 and 2011, mosquitoes were sampled at multiple sites in both Tucson and Nogales, AZ to continue to test these aging techniques as well as our hypothesis that *Ae. aegypti* mosquitoes at the edge of their ecological range do not survive long enough to be efficient vectors of dengue.

## Supporting Information

Figure S1 Expression profiles of seven putative age associated genes. Three to nine replicates of pooled mosquito samples from various time points (1, 3, 5, 10, 15, 20, or 35 days post emergence) were tested for expression profiles of seven previously reported age associated genes. For *AGA011615* (S1D) a significant increase in transcript expression was observed in 35 day old mosquitoes compared to 5-, 10-, and 20-day old mosquitoes. For *AGAP002827* (S1E) a significant increase in expression was observed in one day old mosquitoes. The effect of age on gene expression was tested using a one-way ANOVA followed by Tukey tests. Letters above the bars indicate significant differences in gene expression (p<0.05). If no letters are present no significant differences were observed. Bars indicate standard error.
(TIF)

Figure S2 Average *SCP-1* gene expression per age group. The average *SCP-1* gene expression and standard error of single mosquitoes used in the aging model is presented here by age group, untransformed (n = 154).
(TIF)

Table S1 *Ae. aegypti* orthologues of age associated genes. The five genes (*AGAP009551*, *AGAP011615*, *AGAP002827*, *AGAP005501*, and *AGAP009790*) adopted from Wang, 2010 were transformed to *Ae. aegypti* orthologues with the use of the NCBI Homologene database. The primer sequence and efficiency is provided.
(TIF)
