# Supplementary figures and images for: Aging Field Collected Aedes aegypti to Determine Their Capacity for Dengue Transmission in the Southwestern United States
Source: PLoS One. 2012 Oct 12;7(10):e46946. doi: 10.1371/journal.pone.0046946 (PMC3470585; doi:10.1371/journal.pone.0046946)

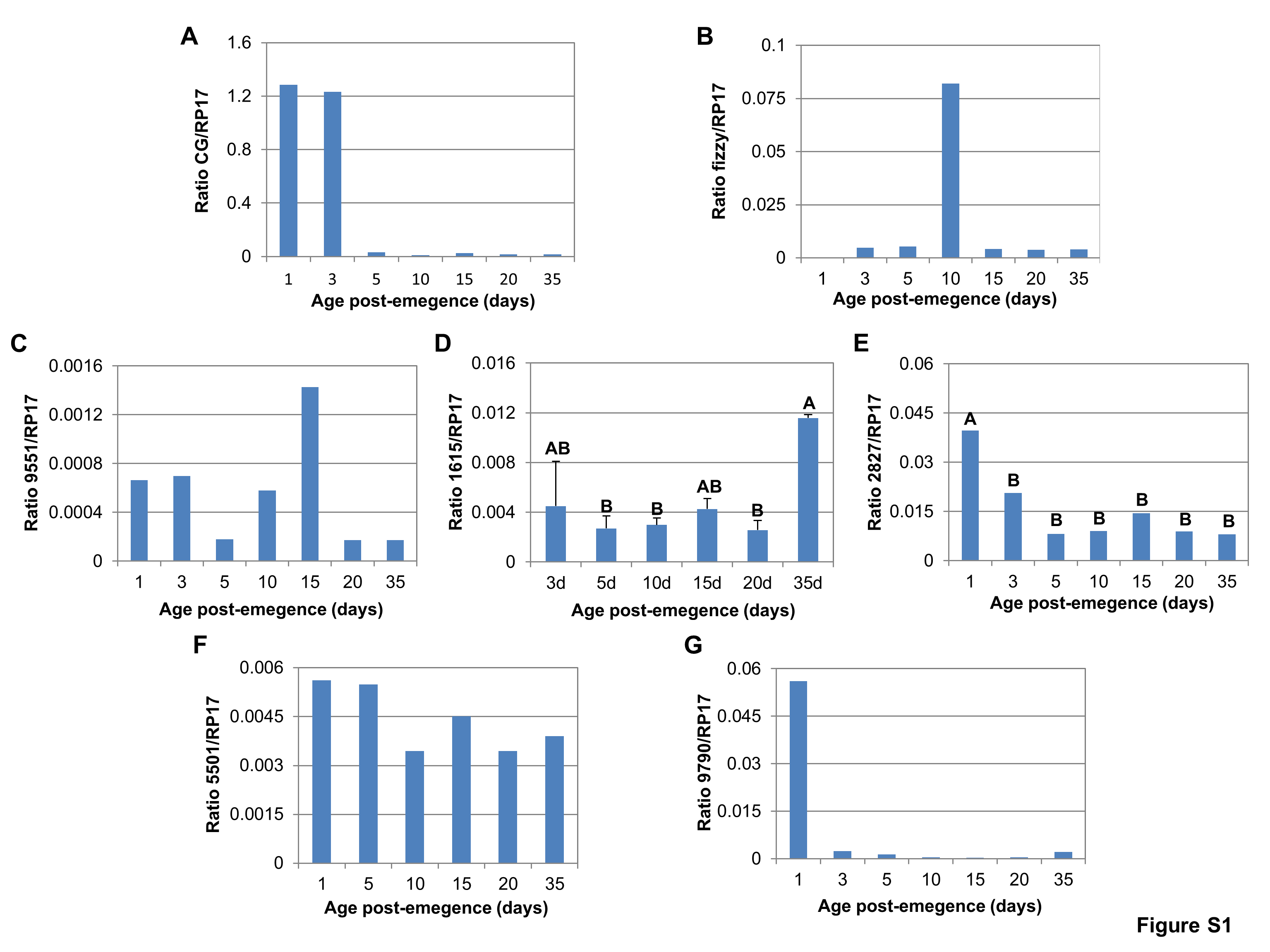

Supplement: Figure S1 — Expression profiles of seven putative age associated genes. Three to nine replicates of pooled mosquito samples from various time points (1, 3, 5, 10, 15, 20, or 35 days post emergence) were tested for expression profiles of seven previously reported age associated genes. For AGA011615 (S1D) a significant increase in transcript expression was observed in 35 day old mosquitoes compared to 5-, 10-, and 20-day old mosquitoes. For AGAP002827 (S1E) a significant increase in expression was observed in one day old mosquitoes. The effect of age on gene expression was tested using a one-way ANOVA followed by Tukey tests. Letters above the bars indicate significant differences in gene expression (p<0.05). If no letters are present no significant differences were observed. Bars indicate standard error. (TIF) [file pone.0046946.s001.tif]

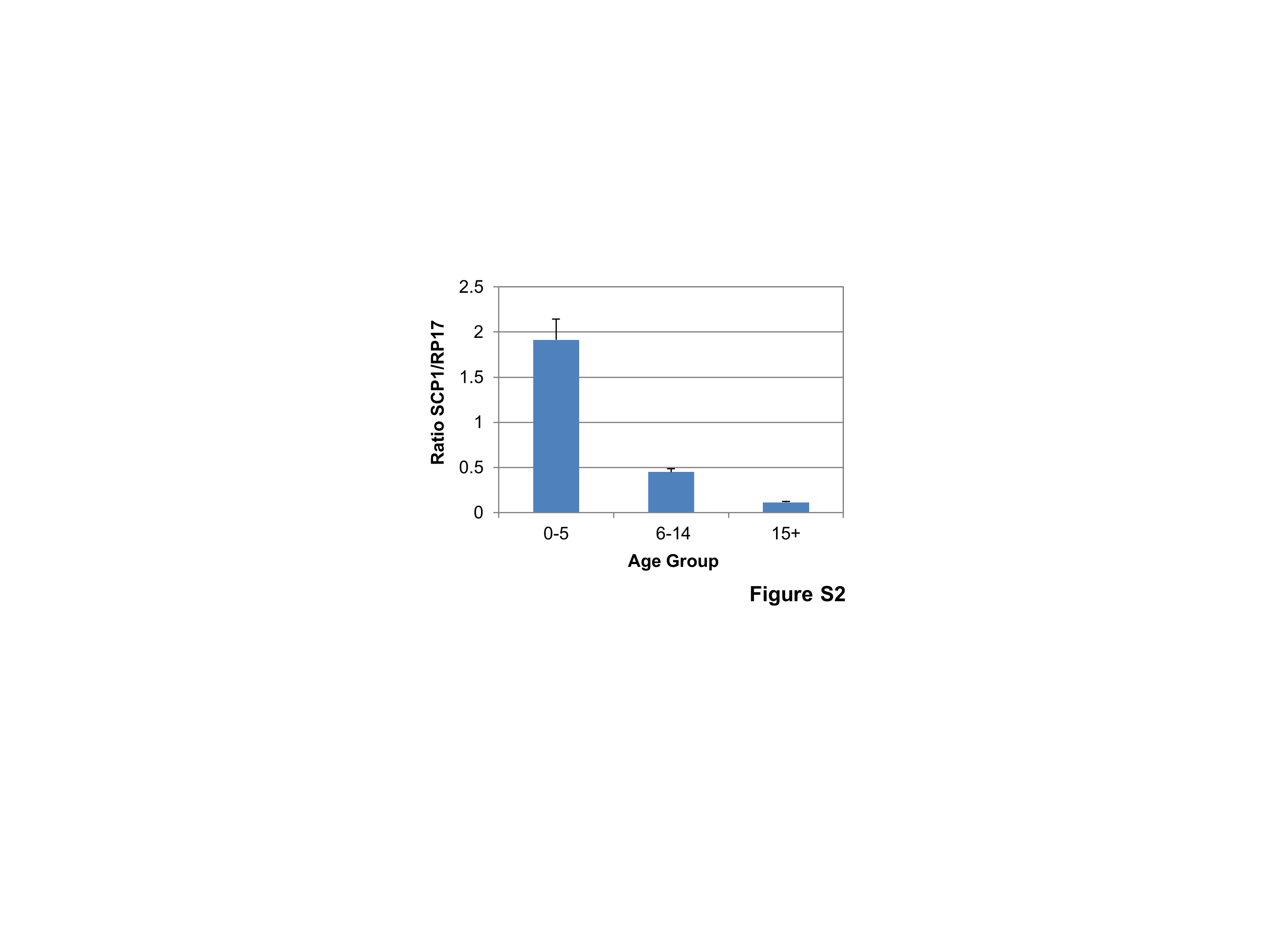

Supplement: Figure S2 — Average SCP-1 gene expression per age group. The average SCP-1 gene expression and standard error of single mosquitoes used in the aging model is presented here by age group, untransformed (n = 154). (TIF) [file pone.0046946.s002.tif]

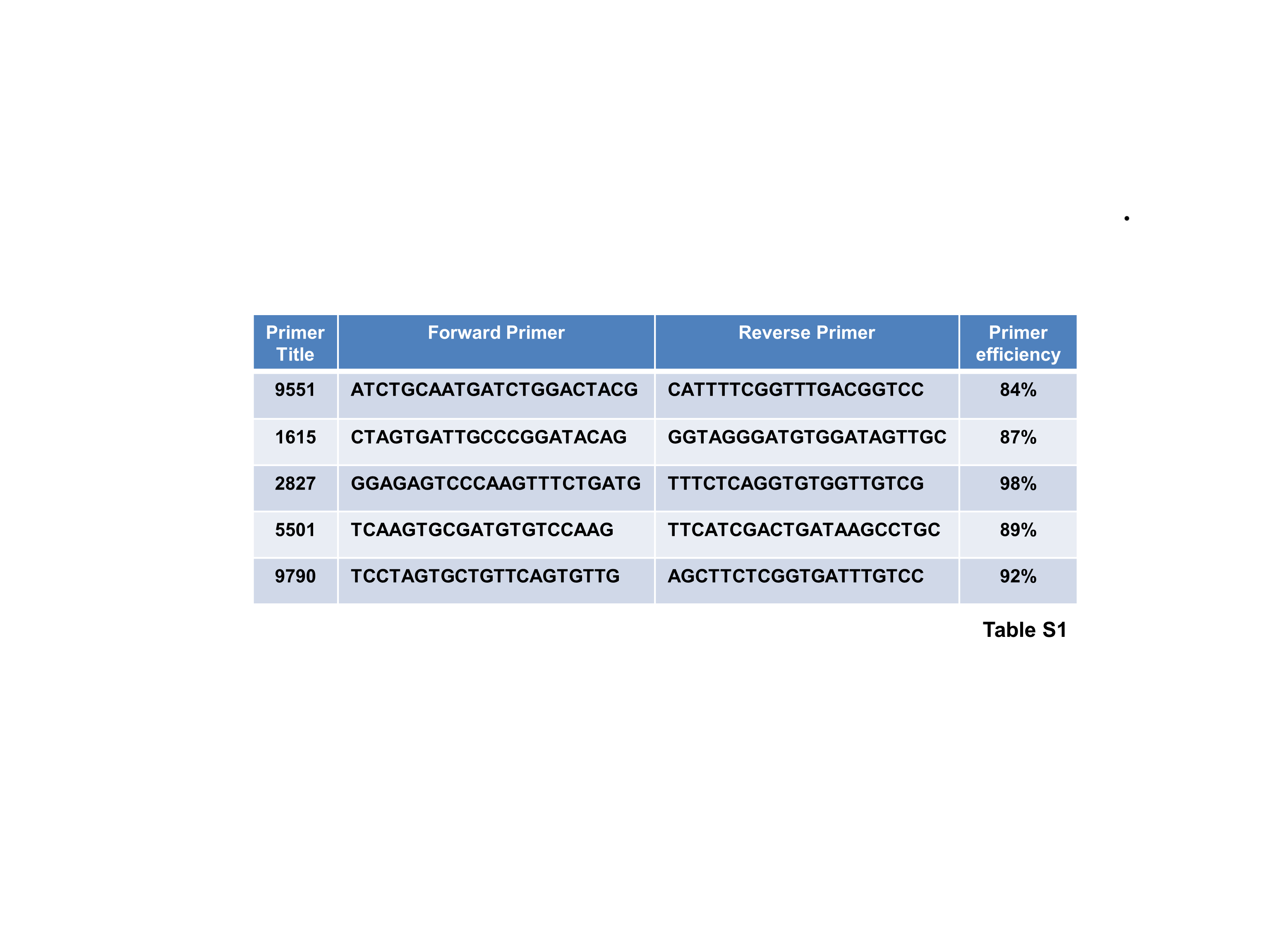

Supplement: Table S1 — Ae. aegypti orthologues of age associated genes. The five genes (AGAP009551, AGAP011615, AGAP002827, AGAP005501, and AGAP009790) adopted from Wang, 2010 were transformed to Ae. aegypti orthologues with the use of the NCBI Homologene database. The primer sequence and efficiency is provided. (TIF) [file pone.0046946.s003.tif]
